# Being a Fair Neighbor—Towards a Psychometric Inventory to Assess Fairness-Related Perceptions of Airports by Residents—Development and Validation of the Aircraft Noise-Related Fairness Inventory (fAIR-In)

**DOI:** 10.3390/ijerph20126113

**Published:** 2023-06-13

**Authors:** Dominik Hauptvogel, Dirk Schreckenberg, Tobias Rothmund, Marie-Therese Schmitz, Susanne Bartels

**Affiliations:** 1 German Aerospace Center, Institute of Aerospace Medicine, Sleep and Human Factors Research, Linder Höhe, 51147 Cologne, Germany; susanne.bartels@dlr.de; 2 ZEUS GmbH, Zentrum für Angewandte Psychologie, Umwelt- und Sozialforschung, Sennbrink 46, 58093 Hagen, Germany; schreckenberg@zeusgmbh.de; 3 Institute for Communication Science, Friedrich-Schiller University Jena, Ernst-Abbe-Platz 8, 07743 Jena, Germany; tobias.rothmund@uni-jena.de; 4 Working Group Biostatistics, Institute of Medical Biometry, Informatics, Epidemiology (IMBIE), University of Bonn, Venusberg-Campus 1, 53127 Bonn, Germany; m.schmitz@imbie.uni-bonn.de

**Keywords:** fairness, justice, procedural, distributive, interactional, informational, aircraft, noise, annoyance, scale, inventory, psychometric, validity, reliability, CFA

## Abstract

Aircraft noise causes a variety of negative health consequences, and annoyance is a central factor mediating stress-related health risks. Non-acoustic factors play an important role in the experience of annoyance where the aspect of fairness is assumed to be a vital component. This paper describes the development of the Aircraft Noise-related Fairness Inventory (fAIR-In) and examines its factorial validity, construct validity and predictive validity. The development of the questionnaire included expert consultations, statements from airport residents and a large-scale online survey around three German airports (N = 1367). Its items cover distributive, procedural, informational and interpersonal fairness. Via mailshot, almost 100,000 flyers were sent out in more (>55 dB(A) L_den_)- and less (≤55 dB(A) L_den_)-aircraft-noise-exposed areas around Cologne-Bonn, Dusseldorf and Dortmund Airport. Thirty-two items were carefully selected considering reliability, theoretical importance and factor loading calculated via exploratory factor analysis (EFA), with all facets achieving high internal consistency (α = 0.89 to 0.92). The factorial validity, analyzed via a confirmatory factor analysis (CFA), revealed that viewing distributive, procedural, informational and interpersonal fairness as distinct factors produced a better fit to the data than other categorizations with fewer factors. The fAIR-In shows adequate results in terms of construct validity and excellent results in terms of the predictive validity of annoyance by aircraft noise (r = −0.53 to r = −0.68), acceptance of airports and air traffic (r = 0.46 to r = 0.59) and willingness to protest (r = −0.28 to r = −0.46). The fAIR-In provides airport managers with a reliable, valid and easy-to-use tool to design, monitor and evaluate efforts to improve the neighborliness between an airport and its residents.

## 1. Introduction

The harmful health effects of aircraft noise have repeatedly been demonstrated in a large number of studies and include a wide range of different effects from annoyance due to noise [1,2], sleep disturbance [3,4], increased risks for cardiovascular diseases [5,6,7], myocardial infarction [8], hypertension [9] and coronary heart disease [6] as well as decreased mental health [10]. Annoyance and disturbance due to noise [1] can be considered as one of the most important effects of noise. A detrimental effect has also been demonstrated in children that relates to health [11], cognition [12] and learning ability, e.g., through a decline in reading and oral comprehension [13].

Even at low noise levels, perceived annoyance around airports is a widespread issue that manifests itself in cognitive, emotional and behavioral aspects [1]. Annoyance is not only understood as an effect of noise, but can also be seen as a mediator between noise exposure and health risks [14]. It has been shown that people who describe themselves as highly annoyed are not only at higher risk for a variety of health issues, e.g., hypertension [15] but also at risk with regard to aspects of mental health such as higher psychological distress [16], depression [17], the use of medication to treat anxiety disorder [18] and a decrease in mental well-being [19].

The psychological model by Stallen [20] suggests that noise annoyance can be seen as a stress response, and the degree of stress depends on the level of perceived control and the capacity to cope with the stressor—that is, noise in this context. Cognitive control has been seen as an important moderator of the impact of noise on behavior and health [21]. The evaluation of sound as unwanted and disturbing results in an activation of the sympathetic nervous system, resulting in a release of stress hormones such as cortisol and an increase in blood pressure [22]. If annoyance is seen as the psychological component of a stress response, it can be hypothesized that annoyance is associated with a variety of other negative stress-related physiological effects. A reduction in annoyance therefore seems to be an essential factor in minimizing the negative health-related consequences of long-term aircraft noise exposure. The evaluation of a sound as noise is highly subjective and depends on a variety of non-acoustic factors, such as attitudes, expectations, and situational and personal factors [2]. As described above, the theoretical model by Stallen [20] suggests that the stress reaction, here the degree of annoyance, depends on the possibility of coping with and control the stressor. Perceptions of psychological control and coping capacity depend, among other things, on how much one perceives trust in the authorities, how predictable such noise events are, whether one has any impact on the noise source, and on whether understandable information about the noise source is provided [20].

Maris et al. [23] were the first to experimentally investigate the role of perceived fairness in the context of aircraft noise research and were able to show that a fair interaction is able to reduce the annoyance caused by noise in a laboratory setting, whereas an unfair procedure tested in an additional study by the same authors [24] increases the annoyance caused by aircraft noise. Fairness can be seen as a major construct underlying many of these non-acoustic factors and has been reviewed in full detail by Hauptvogel et al. [25]. Here, the aspects of distributive, procedural, informational and interpersonal fairness are outlined extensively and were applied to the context of aircraft noise according to theoretical principles.

In summary, the four aspects of fairness in the context of aircraft noise can be described as follows.

Distributive fairness reflects subjective perceptions of fairness in the distribution of resources among groups or individuals. Originally, it was assumed to be based on *equity*, a social comparison in the sense of a cost–benefit ratio [26]. Adapted to the context of aircraft noise research, the *equity* rule suggests that aircraft noise should be distributed in such a way that the ratio between the costs (due to noise pollution) and the benefits that an airport brings to the region is shared equally between all residents. Leventhal [27] introduced the principles of *equality* and *needs* as additional criteria for evaluating distributive fairness. In regard to the equality rule, aircraft noise should be distributed equally, i.e., among as many residents as possible, regardless of other factors (e.g., additional noise exposure from cars). The needs rule would suggest protection of vulnerable residents (e.g., children or elderly residents) from aircraft noise as much as possible, even if others are exposed to more noise as a consequence. As described in the review [25], there is currently no clear answer for which of these distributions elicits the perception of being fairest in the noise-affected residents. Nevertheless, the view of noise as a distributively unfair state offers starting points for interventions to re-establish a balance between cost and benefit or to approach this balance; see [25].

Research on procedural fairness suggests that the process leading to a distribution is at least as important in the experience of fairness as the outcome itself. In the context of aircraft noise, this suggests that the process leading to decisions at the airport is a key determinant in how fairly the distributions of noise are experienced. In their research on procedural fairness, Leventhal [27] developed a set of criteria that constitute a fair process. Applied in the context of noise research [25], these include *representativeness*, which means that the concerns and opinions of all affected parties should be represented at each stage of the decision-making process. The *consistency* rule states that procedures are consistently applied across all residents and times and the *bias suppression* rule specifies that decisions at the airport are not made on the basis of self-interest but rather from an inter-subjective or non-partisan perspective. The *accuracy* rule suggests that decisions are made on the basis of correct and appropriate information and the *correctability* rule proposes that there are also opportunities to revise incorrect or inaccurate decisions. The *ethicality* rule states that processes generally adhere to fundamental ethical and moral standards.

In addition to issues of distributive and procedural fairness, research also indicates that the interaction between the involved parties plays an equally important role in the final perception of fairness [28,29]. This so-called interactional fairness comprises informational fairness on the one hand and interpersonal fairness on the other [30]. It is not sufficient to simply give affected people the opportunity to voice their opinion during the decision-making process; instead, fairness also depends on how the decisions are eventually communicated to these people and how this personal interaction is perceived.

Interpersonal fairness, adapted to the context of aircraft noise research, describes the degree to which aircraft noise-affected residents are approached with respect, dignity and kindness by airport authorities. Informational fairness describes how well decisions regarding aircraft noise are explained to those affected and includes aspects of honesty, transparency and justification. Regarding informational fairness, a number of studies have shown that residents living near airports would like to see more honest, transparent and comprehensive as well as understandable communication in order to establish good neighborliness with the airport [31,32].

As outlined above, individual perceptions of fairness can have an important influence on residents’ attitudes towards the airport and, thus, are assumed to establish a foundation of trust in the long term. A first attempt to develop a psychometric questionnaire of fairness in the context of aircraft noise was undertaken by Bartels [33]. However, due to a very small sample size, further research was needed. Therefore, a reliable and valid measure of perceived fairness in regard to noise distributions at airports is still missing up to now. We aim to fill this gap by developing a measure of perceived distributive, procedural and interactional fairness in the context of airports.

The present paper has three main objectives with three key benefits:

First, the development process of the fAIR-In is presented, which involved item development and a large-scale study with residents of three German airports to validate the instrument. The results allow researchers to further explore the aspect of fairness in relation to noise annoyance and potential other negative health effects of noise.

Secondly, the factorial validity of the questionnaire is examined by comparing one-factor, two-factor, three-factor, and four-factor solutions to test whether the found factor-model of fairness proposed by Colquitt (2001) is also applicable in the context of aircraft noise.

Finally, the construct and the predictive validity of the fAIR-In are investigated to determine whether the fairness facets measured predict residents’ responses to local aviation and aircraft noise management, such as aircraft noise annoyance, acceptance of air traffic and protest behavior against the airport. The results of this analysis provide insight into the effectiveness of the fAIR-In as a comprehensive tool for airport managers, airport authorities and researchers to design, monitor and evaluate efforts to improve relations between residents and the airport.

## 2. Materials and Methods

### 2.1. Sample

We conducted a cross-sectional online study around three German airports in Cologne-Bonn, Dusseldorf and Dortmund. The study procedure was approved by the ethics committee of the Medical Association of North Rhine with the consecutive number 2019235 on 17 May 2021.

These regions were chosen to include airports with different characteristics regarding the number, type and timing of operations. At Cologne-Bonn Airport, night flights with predominantly cargo flights are allowed. In contrast, at Dusseldorf Airport, which is a major German hub airport, night flights are restricted between 10 p.m. and 6 a.m. In contrast to the other two airports, Dortmund Airport is a comparatively small regional airport with a lower number of flights and a ban on regular night flights between 11 p.m. and 6 a.m. Through the choice of airports, different characteristics are covered, providing a comprehensive picture of the perception of fairness under different noise conditions.

The study regions were carefully selected around the airports to include residents experiencing higher aircraft noise exposure (>55 dB(A) L_den_) and residents affected by comparably lower exposure (≤55 dB(A) L_den_). The regions were selected in such a way that aircraft noise was the dominant noise source and both urban and rural regions were represented. Noise exposure was estimated on the basis of the freely available environmental noise maps provided by the state of North Rhine Westphalia from the Ministry for the Environment, Nature Protection and Transport [34]. These freely available noise maps were produced for noise action planning according to the EU Directive 2002/49 on the assessment and management of environmental noise [35].

For the recruitment of participants, we invited participants using mail flyers. Delivery districts were assigned to the identified regions with the help of a mail distribution system, the Postaktuell Manager of the German Post [36]. Flyers containing a link to the survey were sent to every household within these regions.

Two identical questionnaires were programmed in the survey software used, one for the more- and one for the less-exposed areas. Flyers sent to eligible test areas either contained the survey link for the more-exposed areas or for the less-exposed areas. Participants from areas with higher exposure to aircraft noise received exactly the same questionnaire as participants from areas with lower exposure to aircraft noise, but the different links made it possible to assign survey data to the different noise exposure levels.

The flyers contained information about the study and a link to participate. Furthermore, 10 × 100€ were raffled between all participants, which was pointed out on the flyer. Studies have shown that the likelihood to participate is highest when there is a financial incentive and the invitation is framed in a personalized manner that emphasizes the individual importance of each participant’s contribution [37,38]. Participants had the option to take part in the study online through the link provided or to receive a postal version of the questionnaire by calling or e-mailing.

A total of 99,921 flyers were sent out in September and October 2021. Of these, 44,134 were sent to areas with high noise exposure and 55,787 to areas with lower noise exposure. It was assumed that the willingness to participate would be lower in the less noise-exposed areas due to their larger distance from and probably less relation to the airport and its air traffic. Moreover, since exposure is lower, the level of suffering due to noise and the feeling of being affected by (un)fair noise-related decisions were also assumed to be decreased.

A total of 1733 people from the high-exposure areas and 1128 from lower-exposure areas took part. After sorting out discontinued or incomplete entries, there were still a total of 1367 people, 840 from highly exposed regions and 527 from less-affected areas. In total, 1.9% of the more highly aircraft-noise-exposed residents who received a flyer completed the survey and 0.9% of the people who had a lesser exposure to aircraft noise completed the survey. The average completion rate across both noise exposure areas was 1.37% (see Table 1 for an overview).

Separated according to airports, the following pattern emerges.

- Cologne-Bonn Airport: 51,864 flyers sent out, 1721 responses (3.3%) and 819 fully completed survey (1.6%).
- Dusseldorf Airport: 39,794 flyers sent out, 946 responses (2.4%) and 454 fully completed the survey (1.1%).
- Dortmund Airport: 7694 flyers sent out, 194 responses (2.5%) and 91 fully completed the survey (1.2%).

Looking at these distributions, it is evident that there was a very low participation rate across all airports. The slightly higher proportion at Cologne-Bonn Airport could be attributed to the fact that night flights play a special role at this airport that is perceived as particularly annoying.

### 2.2. Fairness Items

The items for measuring fairness-related perceptions in the context of airport management were developed in three different ways. Firstly, a critical incident technique was conducted with scientific experts from the field of aircraft noise research and airport authorities [39]. They were asked about their personal experience in dealing with angry and upset residents and critical incidents in which specific situations were identified. The accusations against the airport and general triggers gave an insight into typical, fairness-related situations that could be used to develop a first set of items. Secondly, the items were derived from research in the literature and existing measurement instruments from other domains, especially organizational psychology [27,40,41]. Finally, affected residents living around airports were interviewed in focus groups, which resulted in further items. These focus groups were conducted as part of the EU project ANIMA investigating, among other things, how a neighborly relationship to the airport can be developed [42].

The classification of facets and subfacets was based on previous research. With regard to the facet of distributive fairness, the subfacets *equity* [26], *equality* [27] and *need* [27] were adopted. For the facet of procedural fairness, the subfacets of *representativeness*, *consistency*, *bias suppression*, *accuracy*, *correctability* and *ethicality* were derived from Leventhal’s research [27]. With regard to the facets of informational and interpersonal fairness, the subfacets of *truthfulness*, *justification*, *respect* and *propriety* were taken from the work of Bies and Moag [41]. *Empowerment* was included as an additional subfacet to the facet of informational fairness. This subfacet reflects whether and how the airport (a) empowers residents to discuss on an equal level, (b) provides contact points for further information, and (c) makes the general information transfer low-threshold. The aspect of empowerment was suggested in the focus groups [42] as a relevant aspect of how residents imagine a fair, neighborly relationship. In the “Vienna Dialogue Forum”, the significance of empowerment as a crucial non-acoustic factor was discussed [43,44]. Empowerment has been classified as a subfacet of informational fairness, as it is mainly reliant on the quality of information regarding comprehensiveness and comprehensibility provided by airports. In contrast to truthfulness and justification, it focuses on enhancing residents’ abilities to express their concerns by providing them with accessible and understandable information on various aspects of the airport and the local air traffic.

A total of 68 items were developed and categorized into the facets of distributive fairness, procedural fairness, informational fairness and interpersonal fairness with their respective subfacets and were randomized in the online questionnaire to minimize sequence effects. The complete questionnaire with instructions and evaluation instructions can be found in the Supplementary Material, both in the original German language (S1.2 of the Supplementary Material) and translated into English (S1.3 of the Supplementary Material).

A pretest with 22 persons was conducted with employees of the German Aerospace Center e.V (DLR). This provided the opportunity to give concrete feedback concerning difficulties in understanding individual items or tasks. By maximizing internal consistency at the subfacet level, a total of 29 of the original 68 items were excluded. Care was taken to exclude redundant items or items that were comparable in regard to their primary statement, as well as items that did not follow the original idea from the literature and/or had high numbers of omitted answers. Another seven items were eliminated after an additional factor analysis in which the factor loadings of items to the respective fairness facet were investigated as described in Section 3.2. A total of 32 items remained in the fAIR-In.

Table 2 provides example items of the final questionnaire representing the four fairness facets (distributive, procedural, informational, and interpersonal fairness) and their subfacets. It is crucial to highlight that while the items were chosen to signify the subfacets (such as equity, equality and need) of their respective fairness facets, it is not asserted that these aspects are measured with sufficient reliability and validity due to the anticipated high correlation among the subfacets. Despite the improvement in measurement economics when reducing the number of items, the loss of insight would be detrimental to the informative value of the fAIR-In.

### 2.3. Scales to Test Construct Validity

Construct validity indicates the extent to which the measured value of a scale is suitable as an indicator for the characteristic that is to be assessed. It therefore describes the validity of a measurement instrument. This means that if the fAIR-In proves to be construct-valid, it truly measures these fairness facets in relation to the airport in the region. According to Campbell and Fiske [45], a distinction should be made between convergent and divergent validity to determine construct validity.

Convergent validity means that correlations are determined between different tests that measure the same or a construct-like construct. The correlation should be as high as possible for a valid test. According to Cohen [46], a small effect is present from r = 0.10, a medium effect from r = 0.30 and a strong effect from r = 0.50.

Divergent validity measures the correlations between different tests and instruments that measure different constructs. Here the correlation should be low or non-existent. According to Cohen [46], a small effect can be considered at r = 0.10.

An overview of all additional scales for measuring construct validity can be seen in Table 3. The response scale of all additional measures was a 5-point scale with the response options (1) not true, (2) a little true, (3) moderately true, (4) quite a bit true and (5) very true. The present response categories were chosen to correspond to an interval level, since the response categories are equally spaced [47,48]. Since there is no established questionnaire that measures fairness in the context of aircraft noise management, no empirically validated relationships could be pre-determined to be tested. For this reason, correlations between the scales and the various fairness facets are calculated and the relationships analyzed.

Trust is an important component of interpersonal interaction. The KUSIV3 scale measures an individual’s generalized expectation of being able to rely on the words and promises in the form of oral or written statements of others or a group [54]. It, thus, describes the individual level of the personality trait of trust in an interpersonal context. For this reason, we expected a positive correlation between interpersonal trust and fairness aspects, especially with interpersonal fairness. A high level of interpersonal trust could lead to residents perceiving the airport and intentions as more positive and therefore lead to residents being more likely to perceive the various fairness criteria as fulfilled.

The political efficacy short scale (PEKS) is used to measure individual political competence and influence beliefs [55] and describes the belief that one can understand and influence political processes [56]. It is considered the most important predictor of political participation. Internal political efficacy, a part of the PEKS, refers to the individual’s self-belief that they have political power to participate [57]. We expected a positive correlation between internal political efficacy and fairness aspects. If residents report high scores in internal political efficacy, then it means that they have more confidence in their ability to actively participate in political decisions and processes. This active, political engagement could lead to actually noticing measures by the airport and, consequently, perceive the airport as fairer.

External political efficacy, the second half of the PEKS, on the other hand, defines the individual’s belief that authorities or systems are susceptible to attempts to influence them. External political efficacy, thus, describes the conviction regarding the political system and is associated with trust in political institutions. If people perceive a generally high external political efficacy, they may also perceive higher values in the fairness aspects, above all with regard to possibilities to interact with airport authorities and to voice their needs and concerns, thus influencing the decisions made by airport authorities.

The USS-8 scale measures the personality trait that describes how one individually experiences and responds to injustice. The construct of injustice sensitivity reflects the disposition of how easily people perceive injustice and how strongly they react to it [58]. These interindividual differences are stable and can be generalized across unjust situations. A distinction is made between four perspectives: the victim, the observer, the beneficiary and the perpetrator perspective. The four perspectives can help explain social phenomena such as the willingness to protest politically, altruism and civility [59]. We expect that people who are more sensitive to injustice will also perceive the airport as less fair. In particular, residents living near the airport with high levels of victim sensitivity might be particularly sensitive to aircraft noise, as they feel they are victims of inequitable noise pollution. The locus of control (IE-4) personality trait derives from social learning [60] and distinguishes between internal and external control beliefs. Internal locus of control describes the extent to which an individual is convinced that they can control events and the extent to which this is experienced as a consequence of their own behavior, whereas external locus of control is associated with viewing events as fate, chance or under the control of powerful others over which they have no influence [61]. We expect a positive relationship between internal locus of control and the different facets of fairness. People with an internal locus of control tend to see themselves as active actors who are able to influence the situation. They might actively participate in participation processes or look for ways to reduce or minimize aircraft noise.

Individuals with an external locus of control, on the other hand, may be more inclined to see themselves as victims of circumstances beyond their control. They may be less committed or more likely to seek compensation or support from others. For this reason, we assume a negative correlation in relation to perceptions of fairness.

Political cynicism can be seen as an attitude and subdimension of political disaffection or disenchantment with politics and is understood as a lack of support for the political system [62]. However, this dimension does not refer to the whole system in general, but describes the skepticism and distrust towards the current political authorities and therefore reflects the opposite of trust in authorities. Individuals with high levels of political cynicism could be skeptical about the airport and its actions, feeling that processes are unfair and that residents’ interests are not adequately considered. For this reason, we expect a negative correlation between political cynicism and the fairness aspects.

However, since none of the scales mentioned above assesses constructs that are exactly the same as fairness or even very similar, all correlations between the mentioned scales and the fairness facets are assumed to be very to rather low (around r = 0.10) according to Cohen’s convention [46].

### 2.4. Criterion Variables to Test Predictive Validity

Predictive validity is the accuracy with which a psychometric questionnaire is able to predict what it is intended to predict [63]. Since this is a cross-sectional study, no directional relationship can be established; therefore, a high correlation between the constructs and the fAIR-In serves as a measure of predictive validity.

Since the fAIR-In is intended to be relevant in the context of aircraft noise research, *aircraft noise annoyance, acceptance of the airport and air traffic, and willingness to protest* are seen as important variables to validate predictive validity, and their relationship with the various fairness facets is examined, as seen in Table 4.

It is assumed that the higher the perceived fairness, the lower the perceived aircraft noise annoyance, as well as the willingness to protest. It is also expected that perceived fairness is positively associated with the acceptance of the airport and air traffic.

To measure predictive validity, scales were used to measure aircraft noise annoyance (5-point ICBEN Question according to ISO/TS 15666) [66]: a scale to measure acceptance of the airport and air traffic and a scale to measure willingness to protest.

The question to assess the noise annoyance is “Thinking about the last 12 months, when you are here at home, how much does noise from aircraft bother, disturb, or annoy you?”.

The acceptance scale was already used in previous studies conducted within DLR [64] and was adjusted to this study. It includes a number of aspects related to the airport and air traffic that participants are asked to assess. Specifically, it asks how necessary, dangerous to human health, unsafe, harmful to the environment, avoidable, bad for air quality, harmful to the climate and reasonable the airport and air traffic are. Here, the mean value of the items was formed after the negatively formulated items were transformed. The internal consistency of this scale is high (α = 0.87). See Table S4 for the complete scale in the original language as well as the English translation. Willingness to protest is also a scale that has already been used in previous internal studies [65] and was adjusted to this study. It was asked if a protest list, petition or similar had been signed, whether contact had been made with the airport or a responsible office to obtain information or to complain, whether a citizens’ initiative against aircraft noise had been joined, whether a demonstration had been attended or whether people had moved to another region because of the aircraft noise or whether a move was being considered. The internal consistency of this set of questions is acceptable (α = 0.70). See Table S5 for the complete scale in the original language as well as the English translation.

### 2.5. Further Questions

To check whether participants were clicking on the answers at random, attention checks were included in the questionnaire. In total, three questions were added, asking participants easy multiple-choice questions, such as which city is the capital of Germany, or choosing between potential results for adding seven plus three.

## 3. Statistical Analysis

### 3.1. Data Cleaning and Preparation

Out of a total of 2872 participants who started the questionnaire, 1505 incomplete datasets were excluded from further analyses (52.40%). Of these, 1406 of the 1505 incomplete datasets were individuals who quit the questionnaire before completing it and did not save their answers.

All items of the questionnaire were mandatory questions. This means that one could not continue the questionnaire if a question was skipped. The fairness questions, on the other hand, were designed in such a way that it was possible to skip individual items. If this happened for one of the fairness questions, the participants were informed that one or more answers were missing and had to confirm that this was intentional. Otherwise, they had the opportunity to re-enter missing answers in the fairness items.

Among the 99 people who completed the questionnaire but had missing values in the fairness questions, 26 people had to be excluded. The criterion for this was omission of more than half of the items per subfacet (e.g., equity, equality). Another 73 people who had less than half of the missing items per subfacet were also eliminated for the subsequent calculations, as the maximum likelihood estimation method for the confirmatory factor analysis can only handle complete datasets [67]. However, they were included in the selection of final items to help discard items that had similar characteristics. This led to a total of 1367 complete datasets (47.64%).

The dataset of fully completed questionnaires was randomly divided into two separate parts. The two separate datasets (Dataset A and Dataset B) could, thus, be used for independent calculations and prevent overfitting [68].

### 3.2. Item Selection

Item selection was performed in several steps within Dataset A. First, scales were created for the respective fairness facets (distributive, procedural, informational and interpersonal fairness) and the respective subfacets (e.g., equity, equality, need for distributive fairness and process and decision control, etc., for procedural fairness) according to the most recent fairness literature; see [69]. A separate reliability analysis was carried out for each subfacet and further items were excluded, which resulted in an increase in the internal consistency of the scale. This was performed for each subfacet until no more improvement could be achieved. Both Cronbach’s alpha and McDonald’s omega were calculated to check internal consistency. McDonald’s omega (ω) values are more robust and recent research shows that these values should be reported rather than Cronbach’s alpha (α) [70]. However, since no significant differences were found, the traditional Cronbach’s alpha (α) is reported below.

Generally, when excluding items, attention was also paid to the item difficulty in order to minimize floor or ceiling effects [71].

After aiming for a number of two to three items per subfacet, in order to reduce the redundancy and enhance the efficiency of the questionnaire, a factor analysis was calculated. Additional items were removed according to three criteria postulated by Tabachnick (2007) [72,73]. The first criterion suggests the exclusion of items with a factor loading below 0.32 on any factor. Secondly, items were removed that had loadings exceeding 0.32 on more than one factor. Lastly, it was ensured that factors had at least five items with factor loadings over 0.5. Only highly relevant items were retained in the final fAIR-In, even if they did not meet these criteria. At the end, the fAIR-In included 32 items.

### 3.3. Factorial Validity

In order to test factorial validity, we calculated confirmatory factor analyses (CFAs) using Dataset B. The “lavaan” package was used in RStudio (Version 1.3.959) for this purpose [67]. This package provides robust estimates with Santorra–Bentler correction for the maximum likelihood (ML) procedure. The ML estimation with Santorra–Bentler correction should be preferred to other estimators that can handle non-normal data, such as the asymptotically distribution-free estimator (ADF) [74]. ML is superior to other estimators because it is more stable, more accurate and has a higher precision in terms of empirical and theoretical fit [75].

Four different models were compared to each other as seen in Table 5. *Model 1*, the four-factor model, distinguishes between distributive, procedural, informational and interpersonal fairness as proposed by Greenberg [29]. *Model 2*, the three-factor model, distinguishes between distributive, procedural and interactional fairness, subsuming the aspects of informational and interpersonal fairness under one factor as suggested by Bies and Moag [41]. In *Model 3*, the two-factor model, distributive fairness and procedural fairness are assumed, in which the aspects of informational and interpersonal fairness are combined into the procedural fairness as seen in Niehoff and Moorman [76]. *Model 4*, the one-factor model, comprises all items under the aspect of a superordinate perception of fairness, as researched by Colquitt [69].

Since some subfacets contained only a few items and, even more relevantly, correlated very strongly with each other in some cases, they were not modeled separately in an additional level within the confirmatory factor analysis.

## 4. Results

### 4.1. Sample

Table 6 describes the sample. A total of 1367 complete data sets were collected. The randomized division of the complete data set into data set A and data set B shows that the characteristics of the sample are equally represented in both parts and that they do not differ noticeably from each other. The educational levels surveyed based on the German education system were converted to the international standard classification of education (ISCED-2011) [77]. An overview of the sampling characteristics for the various airports can be found in Supplementary Material (Table S1).

### 4.2. Item Selection

The final 32 items on distributive fairness, procedural fairness, informational fairness and interpersonal fairness have high internal consistency as Cronbach’s alpha (α) ranges from 0.89 to 0.92, as seen in Table 7. The final questionnaire, as well as instructions for assessment and the categorization of the items into the various facets, can be found in the Supplementary Material, both in the original German language (S1.2 of the Supplementary Material) and translated into English (S1.3 of the Supplementary Material). Furthermore, a classification of items within the subfacets can be seen in Supplementary Material S1.4.

### 4.3. Validity

#### 4.3.1. Factorial Validity

As described in Section 3.2, four different models were computed in which fairness was described by four, three, two or one factor.

The results, seen in Table 8, show that the best-fitting model is Model 1, differentiating between four factors. The worst-fitting model is Model 4, the model with only one factor. To determine whether the Models also differ significantly from each other, the likelihood ratio (LR) test was used, which showed a significant result each time the Models were compared. This means that Model 3 is significantly better than Model 4, Model 2 is significantly superior to Model 3 and Model 1 is significantly better than Model 2. The results, seen in Table 8, are in line with the work of Colquitt [69] and suggest that a conceptualization of fairness into four distinct facets is advisable and statically superior to other types of conceptualizations in the field of aircraft noise research.

#### 4.3.2. Construct Validity

Table 9 shows all correlations between the different fairness facets and the other constructs, as described in Section 2.3. There is no statistically significant correlation between interpersonal trust and the fairness facets (r = −0.02 to r = 0.06). The correlation between internal political efficacy and the fairness facets is small but significant for procedural fairness (r = −0.10) and for interpersonal fairness (r = −0.09). External political efficacy is more substantially and statistically significantly correlated with all fairness facets (r = 0.20 to r = 0.29). For the scales on injustice sensitivity, the results are mixed. There is a small significantly positive correlation between victim sensitivity and distributive fairness (r = 0.18), interpersonal fairness (r = 0.14) and procedural fairness (r = 0.11). We also find a small significantly negative correlation between procedural fairness and both observer sensitivity (r = −0.11) and perpetrator sensitivity (r = −0.11). Internal control perception correlates slightly but statistically significantly with procedural, informational and interpersonal fairness (r = 10 to r = 0.12).

External control perception does not correlate with any of the fairness facets.

Political cynicism correlates slightly but statistically negatively with the fairness facets (r = −0.23 to r = −0.10). We also calculated the results separately according to noise exposure, whereupon it turned out that there are no systematic differences. An overview can be found in the Supplementary Material (Table S2).

#### 4.3.3. Predictive Validity

Table 10 shows all correlations between the fairness facets and the criterion variables. As hypothesized, Table 10 shows that all fairness facets are negatively related to annoyance (r = −0.53 to r = −0.68), positively connected to acceptance of airport and air traffic (r = 0.46 to r = 0.59) and negatively correlated to willingness to protest (r = −0.28 to r = −0.46). All correlations are statistically significant.

## 5. Discussion

The present work had three central objectives. First, we outlined the process of item development. We generated items in different ways, including a search of the literature, expert interviews and focus groups. Then, we identified good items based on their measurement properties with statistical analyses using a large-scale online survey of airport residents.

Secondly, we examined the factorial validity of the questionnaire. A confirmatory factor analysis indicated that a classification into the four fairness facets of distributive, procedural, informational and interpersonal is superior to other categorizations of fairness in the context of aircraft noise research. This four-factor model achieved good model fit values and, thus, confirms the factorial validity of the questionnaire. As mentioned before, there has long been disagreement about the dimensionality of fairness questionnaires. In an organizational context, Colquitt [69] was able to show that the four-factor structure is superior to other factor structures. In the context of aircraft noise, we can replicate these findings. A model describing fairness as four factors provides a significantly better fit to the data compared to alternative models also in the context of aircraft noise research.

The third aim of this study was to investigate the construct and predictive validity of the instrument. Regarding construct validation, it is worth noting that the correlations between fairness facets and the corresponding correlates were consistently small to moderate. This finding is actually favorable, as excessively high correlations would undermine the specificity of the instrument. The results indicate that the fAIR-In measures constructs that are distinct from general interpersonal trust or injustice sensitivity, further confirming its construct validity. Our results suggest that the fAIR-In is an independent measurement instrument that does not assess stable personality traits, but instead captures specific aspects of the perception of airport management.

The very low correlations between interpersonal trust and the fairness facets indicate that the scales measure different things. Admittedly, we expected that people with a high score on this variable would also have higher perceived fairness aspects in relation to the airport. The low correlation could be explained by the fact that the fAIR-In does not measure general expectations, but instead specific circumstances.

Internal political efficacy [50] correlates slightly with the fairness facets, but in particular, significantly negatively with procedural fairness. We had assumed a positive correlation on the assumption that people with high scores participate more actively in decision-making processes. The negative correlation could arise if these people, despite their willingness, notice that there are no or hardly any opportunities for participation.

External political efficacy [50] correlates significantly positively with all fairness facets. We conclude that people who generally see possibilities to influence authorities and who report a stronger belief in authorities’ intention to consider the concerns of the population are more prone to also perceive a higher fairness in the distribution of aircraft noise, the decision-making procedures coming to this distribution and the information and interaction connected with it.

As suspected, there is little to no correlation between injustice sensitivity and the fairness facets. Unexpectedly, however, there is a small but significant positive correlation between victim sensitivity and the fairness facets [51]. This means that residents that are more sensitive to injustice in regard to their own disadvantages judge the fairness of the aircraft noise distribution, the procedural aspects of airport management and interpersonal aspects higher. In general, it could be argued that people with increased sensitivity to injustice are more aware of topics related to injustice. Being more sensitive to injustice, these individuals may be more attentive of the airport’s efforts and may have been in contact with the airport, resulting in a more positive image. As a result, individuals may be more likely to view fairness aspects of the airport as positive, while individuals with lower injustice sensitivity may have formed their opinions and lack the intrinsic drive to convince themselves otherwise.

With observer sensitivity, on the other hand, the opposite could be the case, namely that people who are not as affected themselves compare themselves with other residents who are more affected by aircraft noise. This perceived difference could lead to the airport being perceived as less equitable.

A significant positive correlation was found between internal locus of control and the fairness facets for procedural, informational and interpersonal fairness. As assumed, this could be due to the fact that people with high values do not consider themselves helpless and actively seek opportunities for participation.

External locus of control correlates only very slightly and not significantly with the fairness facets of the fAIR-In. Since this perception is not supposed to be captured by the fAIR-In, this result can be interpreted as an indication of divergent validity. Thus, a high correlation would indicate that the fAIR-In does not describe people’s perception of the airport in the region, but rather the tendency to have no decision-making power anyway, regardless of circumstances.

Unlike external political efficacy, political cynicism does not refer to the entire political institution, but describes the skepticism and distrust of the current political authorities. With a negative correlation varying between r = −0.10 and r = −0.23, it can be assumed that this negative correlation is also evident. We conclude from these results that people who generally question trustworthiness of (current) political authorities are also more likely to mistrust airport authorities. Thus, indications exist that the fAIR-In really does measure aspects related to (mis-)trust in authorities.

However, in sum, all of the considered scales comprise rather divergent constructs instead of convergent constructs. Attempts should be made in future research to identify contracts that can serve as scales for convergent validation in order to further test the construct validity of the newly developed fAIR-In.

In regard to predictive validity, all hypotheses were confirmed. The correlations found between all fairness facets and the predicted variables of annoyance, acceptance and willingness to protest are very high, suggesting that all four facets are relevant predictors.

Perceived airport fairness is negatively related to annoyance and positively related to (a) the acceptance of the airport and air traffic and (b) the willingness to protest. These are exactly the relationships that were predicted and show that the fAIR-In is able to measure practically relevant aspects of airport management, making it a useful evaluation tool. The relationships between fairness and other factors found here can be embedded in the context of research on non-acoustic factors, in particular the model proposed by Stallen [20] that considers annoyance as a stress response to noise. From the perspective of this theoretical model, the perception of fair procedures, information and behavior of airport authorities can serve as a source for control and the capacity to cope with the noise.

The comparatively low correlations between fairness perceptions and the willingness to protest may also be explainable. Whether or not a person shows protest behavior depends on various factors including personality, situational and cultural aspects—even when the perceived unfairness is high. Similarly, as pointed out before (e.g., [78]), the number of complaints about aircraft noise does not reflect the degree of noise annoyance around airports to the full extent. According to the authors, complaining behavior results from an interaction between many personal and environmental factors and not only annoyance due to noise. The fact that protest behavior is the result of a variety of different influencing factors explains why fairness is not as strongly associated in comparison to annoyance and the acceptance of the airport and air travel. In subsequent work, a multiple regression analysis or a structural equation model should be carried out to further consider the respective correlations and intercorrelations and to further elaborate the specific influences of the respective fairness facets.

The results found with regard to the strong relationship between fairness and other aircraft-noise-induced responses such as annoyance, acceptance and willingness to protest can only be embedded in current research to a limited extent, as little empirical research is available at this point in time. However, the present results support the findings from a laboratory study approach by Maris and colleagues [23,24] suggesting that providing fair procedures by giving voice and process control to the noise-affected individual reduces noise-induced annoyance whilst unfair procedures (i.e., ignoring stated preferences) increase annoyance. While Maris et al. examined the effect of procedural fairness on annoyance, the present study goes beyond, as it also shows a strong association among distributive, interpersonal and informational fairness and annoyance. To this end, the present findings confirm the assumptions of Stallen’s theoretical framework [20] according to which a fair exchange between the airport and its residents that includes giving information and justification helps affected residents cope with the noise. A central point in this theoretical framework is the assumption that providing relevant information enhances the foreseeability of noise and, thus, increases residents’ perceived control over the noise situation.

Especially in times of (operational) changes at the airport, which are connected with changing noise levels, decisions at the airport and their justification play a major role in the acceptance of outcomes.

The present findings suggest that aspects of distributive, procedural, informational, and interpersonal fairness are of great relevance to how residents react to these processes and final decisions and how annoyed residents eventually are. In addition to annoyance, the present work also provides evidence that fairness has an influence on the acceptance of the airport and air traffic, as well as the willingness of residents to protest.

The correlations found point at new scopes for further analysis of inter-relationships. Since the development of the fAIR-In was the main focus of this paper, it was not possible to go further into these associations and possible moderation and mediation effects. In a future publication, these relationships will be analyzed and discussed within the framework of a comprehensive structural equation model.

In the future, these relationships could be analyzed in depth and, thus, expand the current understanding of non-acoustic factors in connection with fairness facets in the context of aircraft noise management.

## 6. Limitations

One major limitation that needs to be mentioned was that, due to the COVID-19 pandemic, air traffic has decreased considerably since the beginning of 2020; however, this was not included in the survey questions. For example, the ICBEN question on noise annoyance asked how disturbed or bothered people felt in the last 12 months. Since the survey was conducted at the end of 2021, the actual extent of noise annoyance and, thus, also potentially the fairness-related perception might not have been correctly represented. During the study and through telephone contact with survey participants, comments were made that air traffic was reduced so much during the COVID-19 pandemic that there was currently hardly any annoyance due to aircraft. The participants pointed out that this was due to the current situation and that they also expected that once air traffic increased again, the nuisance would also increase again. For this reason, the measurement of annoyance and related aspects such as perceived fairness may be somewhat biased. However, as the majority of respondents stated that they live in their own home, it can be assumed that aspects of attitudes towards the airport have existed for some time and are therefore unlikely to change as a result of the reduction in flights. However, the circumstances of the survey within the COVID-19 pandemic warrant further investigations and replications of the study results in future surveys. Furthermore, future studies should capture the duration of residence, as this can influence attitudes towards the airport and the perceptions of fairness aspects. Unfortunately, this was not included in the present study.

Due to the focus of the manuscript on the development and validation of the fAIR-In, noise exposure was not included in the standard calculations. However, it can be seen (see Supplementary Material Tables S2 and S3) that there are no significant differences between the higher- and lower-exposure groups of residents in terms of the results. These results also emphasize yet again the relevance of the fAIR-In. This shows that fairness does not only play a role for highly exposed people, but that fairness also has a significant influence on annoyance, acceptance of the airport and air traffic, and the willingness to protest at comparatively low levels of aircraft noise exposure. Furthermore, in this study, the method of sending out flyers according to postal codes that were matched with noise contour maps did not allow for a higher resolution of the exact noise exposure. In future studies, a more precise differentiation between different noise categories could be achieved by targeting the respondents who are located in specific areas, specifically areas with noise levels over 65 dB(A) L_den_. Another limitation of the study concerns the selection of participants. Although the flyers were distributed to all households in the selected areas, participation was not random but rather self-selected. Thus, it cannot be ruled out that a self-selection bias came into play in the sample. For example, people who describe themselves as highly annoyed are associated with a higher level of suffering and, thus, also a desire to change something about the current situation. It can therefore be assumed that the level of aircraft noise annoyance in this sample is higher compared to the general population in the region. Nevertheless, the results of the present work are to be considered important, as they allow the foundation for further research on fairness aspects in the context of aircraft noise.

As this study is a cross-sectional study, no causality can be concluded. It is therefore not clear whether people who feel that they are treated more fairly by the airport are less annoyed, or whether people who feel less annoyed perceive the airport to act more fairly. Drawing on the work of Maris et al. [23], who found a causal relationship between fairness and annoyance, it could be argued that the direction of the relationship is from fairness to annoyance. However, obtaining further longitudinal data on fairness perceptions on the one hand and noise responses such as annoyance, acceptance of airport and air traffic as well as willingness to protest on the other is highly recommended in future noise surveys and epidemiological examinations.

## 7. Practical Implementation of the fAIR-In in Airport Regions

The present paper introduces the fAIR-In, an empirically validated psychometric instrument that may drive future research in the field of aircraft noise. Furthermore, the aim was to provide a tool to assess the relationship between an airport and its residents and therefore serve as a foundation for subsequent measures to address any existing concern and improve the relationship, that is, lead to a fairer and more trustful relationship between airport operators and residents in the long term.

With regard to the implementation of interventions in the context of airport management, the fAIR-In is in a unique position to provide essential support.

For example, the implementation of the fAIR-In around the airport can provide evidence as to which aspects of fairness are perceived particularly well or especially negatively. This enables targeted and efficient interventions to be planned with the aim of increasing the perceived fairness and building a neighborly relationship with the airport. Furthermore, it is also possible to evaluate implemented interventions in the sense of a pre-post comparison. Most of the airport’s interventions are implemented and expected to have the desired effect. However, it turns out that hardly any evaluation of the activities is carried out [43]. Nevertheless, this does not allow any conclusions to be drawn about whether the intervention was useful and truly effective. Additionally, it is not possible to make comparisons between different airports and to empirically determine the benefits of the measures implemented. The fAIR-In offers a low-cost, quick-to-implement tool to help airports close this important gap.

Since the primary issue with aircraft noise is that it is man-made noise, unlike natural sound sources, aspects play a role here that can also be applied to other scenarios. Noise sources that will become increasingly relevant in the future, such as noise from wind turbines, heat pumps, or even drones or air taxi noise, can cause annoyance among residents. Therefore, an early integration of fairness in planning procedures is relevant to minimize the negative consequences of noise for residents.

## 8. Conclusions

This study establishes the effectiveness of the fAIR-In as a psychometric instrument for evaluating fairness-related dimensions in airport and noise management. Results on the validity of the inventory suggest that the four fairness facets that were derived in an organizational and juridical research context (distributive, procedural, informational and interpersonal fairness) can be replicated in the context of aircraft noise exposure and its management by the airport. These four fairness facets can be obtained with a high reliability in terms of internal consistency. The fAIR-In is capable of measuring aspects that characterize more or less fair distributions, procedures and interactions instead of mere personality traits. Furthermore, the fAIR-In demonstrates strong predictive power regarding important consequences of unwanted exposure to aircraft noise including noise annoyance, airport acceptance and willingness to engage in protest. As a result, airport managers can rely on the fAIR-In as a reliable, valid and practical tool. By utilizing the fAIR-In, airport managers can implement targeted interventions, monitor progress and evaluate outcomes, thus facilitating the development of long-term improvements in the relationship between airports and neighboring residents.

## Figures and Tables

**Table 1:** Overview of the response rate as well as the cleaned complete responses, classified according to noise exposure and in total.

	Higher Noise Exposure	Lower Noise Exposure	Total
Response rate	3.93%	2.02%	1.73%
Completion rate	1.90%	0.90%	1.37%

**Table 2:** Examples of items created to measure fairness-related perceptions in relation to airport management.

Fairness Facet	Subfacet	Item Example
Distributive fairness	Equity	The airport brings me more advantages than disadvantages.
	Equality	Due to the different approach and departure directions of the aircraft, the noise pollution is evenly distributed among the residents.
	Need	The approach and departure directions are set in such a way that those in need of protection, such as children or sick people, are affected as little as possible by aircraft noise.
Procedural fairness	Process control	Before decisions are made on aircraft noise, I have the opportunity to make my views known to those responsible.
	Decision control	When decisions are made about aircraft noise, I can influence the outcome of the decision-making process.
	Bias suppression	The airport attempts to make decisions in an unbiased and neutral manner.
	Representativeness	All parties who are affected are involved in decisions relevant to aircraft noise.
	Consistency	Residents cannot understand why different rules apply at different airports, e.g., on night rest times or flight bans.
	Accuracy	In the decision-making processes, those responsible often make decisions on the basis of incorrect information.
	Correctability	I have the possibility to take action against decisions that I think are wrong.
Informational fairness	Truthfulness	The airport is honest about its plans for the future.
	Justification	The airport explains and justifies decisions relevant to aircraft noise in detail.
	Empowerment	The airport provides information that enables residents to discuss with airport authorities on an equal footing.
Interpersonal fairness	Propriety	The airport strives for an exchange with noise-affected residents that is conducted at eye level.
	Respect	The exchange between the airport and local residents is respectful.

Note: The response scale was a 5-point scale with the response options (1) not true, (2) a little true, (3) moderately true, (4) quite a bit true and (5) very true.

**Table 3:** Overview of all additional measures included in the survey to test construct validity.

Scale	Source
Interpersonal trust (KUSIV3)	[49]
Political confidence and influence perception (PEKS)	[50]
Sensitivity to injustice (USS-8)	[51]
Perception of control (IE-4)	[52]
Political cynicism (KPZ)	[53]

**Table 4:** Overview of all additional measures included in the survey to test predictive validity.

Scale	Source
Aircraft noise annoyance	[47]
Acceptance of airport and air travel	Adjusted from [64]
Protest behavior	Adjusted from [65]

**Table 5:** Facet division depending on model.

Model	Facets
Model 1 (4-factor solution)	Distributive, procedural, informational, interpersonal
Model 2 (3-factor solution)	Distributive, procedural, interactional
Model 3 (2-factor solution)	Distributive, procedural
Model 4 (1-factor solution)	Overall fairness

**Table 6:** Sample description for the complete sample and the two randomly divided halves. The percentages are reported in the brackets.

	Dataset A	Dataset B	Total
Total	691	676	1367
	N (%)	N (%)	N (%)
Gender			
Male	409 (59.2)	399 (59)	808 (59.1)
Female	279 (40.4)	271 (40.1)	550 (40.2)
Diverse	3 (0.4)	6 (0.9)	9 (0.7)
Age			
18–24	28 (4.1)	23 (3.4)	51 (3.7)
25–34	95 (13.7)	108 (16)	203 (14.9)
35–44	123 (17.8)	121 (17.9)	244 (17.8)
45–54	114 (16.5)	135 (20)	249 (18.2)
55–64	188 (27.2)	172 (25.4)	360 (26.3)
65–74	95 (13.7)	86 (12.7)	181 (13.2)
75–84	41 (5.9)	29 (4.3)	70 (5.1)
≥85	7 (1)	2 (0.3)	9 (0.7)
Education			
Still in school	5 (0.7)	2 (0.3)	7 (0.5)
Primary education	29 (4.2)	26 (3.8)	55 (4)
Lower secondary education	113 (16.4)	112 (16.6)	225 (16.5)
Upper secondary education	544 (78.7)	536 (79.3)	1080 (79)
Living conditions			
Renter	212 (30.7)	254 (37.6)	466 (34.1)
Property owner	479 (69.3)	422 (62.4)	907 (65.9)
Job connected to airport			
Direct	17 (2.5)	13 (1.9)	30 (2.2)
Indirect	25 (3.6)	17 (2.5)	42 (3.1)
Not connected	649 (93.9)	646 (95.6)	1296 (94.7)
Airport in vicinity			
Cologne-Bonn Airport	418 (60.5)	401 (59.3)	819 (59.9)
Dortmund Airport	50 (7.2)	41 (6.1)	91 (6.7)
Dusseldorf Airport	222 (32.1)	232 (34.3)	454 (33.2)
Noise exposure			
High exposure (>55 dB(A) L_den_)	430 (62.2)	410 (60.7)	840 (61.4)
Low exposure (≤55 dB(A) L_den_)	261 (37.8)	266 (39.3)	527 (38.6)
Participation			
Online	658 (95.2)	650 (96.2)	1308 (95.7)
Paper–pencil	33 (4.8)	26 (3.8)	59 (4.3)

**Table 7:** Overview of the internal consistency of the different fairness facets of the fAIR-In.

Fairness Facets	Number of Items	Cronbach’s Alpha (α)
Distributive fairness	7	0.89
Procedural fairness	13	0.90
Informational fairness	7	0.89
Interpersonal fairness	5	0.92
Total	32	0.96

**Table 8:** Comparison of a priori fairness factor structures in the context of aircraft noise research.

Model	Structure	χ^2^ (Robust)	Df	χ^2^/df	CFI (Robust	RMSEA (Robust)	RMSEA—CI	SRMS	AIC	LR-Test
1	4-factor	1286.49 ***	458	2809	0.931	0.055	0.052–0.059	0.046	52,433.460	
2	3-factor	1400.83 ***	461	3039	0.922	0.059	0.055–0.063	0.047	52,567.217	Model 1 vs. Model 2 ***
3	2-factor	1664.0 8 ***	463	3594	0.899	0.067	0.063–0.070	0.050	52,881.206	Model 2 vs. Model 3 ***
4	1-factor	2198.29 ***	464	4738	0.853	0.081	0.077–0.084	0.058	53,524.399	Model 3 vs. Model 4 ***

Note: CFI: comparative fit index; TLI: Tucker–Lewis index; RMSEA: root-mean-square error of approximation; LR test: likelihood ratio test. (***: *p* ≤ 0.001).

**Table 9:** Pearson r correlations between the individual fairness facets of the fAIR-In and all additional scales for testing construct validity. (**: *p* ≤ 0.01, *: *p* ≤ 0.05).

Constructs	Distributive Fairness	Procedural Fairness	Informational Fairness	Interpersonal Fairness
Interpersonal trust (KUSIV3)	−0.02	0.02	0.05	0.06
Political efficacy (PEKS)				
Internal political efficacy	−0.06	−0.10 **	0.02	−0.09 *
External political efficacy	0.20 **	0.25 **	0.29 **	0.29 **
Injustice sensitivity (USS-8)				
Victim sensitivity	0.18 **	0.11**	0.06	0.14 **
Observer sensitivity	−0.05	−0.11**	−0.06	−0.01
Beneficiary sensitivity	0.02	−0.02	−0.05	−0.01
Perpetrator sensitivity	−0.09 *	−0.11 **	−0.07	−0.06
Control perception (IE-4)				
Internal control perception	0.07	0.10*	0.12 **	0.12 **
External control perception	0.03	0.02	−0.03	−0.02
Political cynicism (KPZ)	−0.10 **	−0.19 **	−0.23 **	−0.21 **

**Table 10:** Pearson r correlation between the different facets of the fAIR-In and the predictive variables. (**: *p* ≤ 0.01).

Construct	Distributive Fairness	Procedural Fairness	Informational Fairness	Interpersonal Fairness
Annoyance	−0.68 **	−0.61 **	−0.53 **	−0.60 **
Acceptance	0.59 **	0.53 **	0.46 **	0.51 **
Willingness to protest	−0.46 **	−0.36 **	−0.28 **	−0.42 **

## Data Availability

The data presented in this study are available on request from the corresponding author.

## Supplementary Materials

The following supporting information can be downloaded at: https://www.mdpi.com/article/10.3390/ijerph20126113/s1, Table S1: Sample description according to the airport in the vicinity; Table S2: Pearson r correlations between the individual fairness facets of the fAIR-In and all additional scales for testing construct validity separated according to the different degrees of noise exposure; Section S1.2: fAIR-In Questionnaire in German with original items; Section S1.3: fAIR-In Questionnaire translated into English; Section S1.4: fAIR-In Items in German and English with classification to facts and subfacets; Table S4: Questionnaire used in this study to measure acceptance of the airport and air travel; Table S5: Questionnaire used in this study to measure protest behaviour. Reference [79] is cited in the supplementary materials.
