# Supplementary material for: Being a Fair Neighbor—Towards a Psychometric Inventory to Assess Fairness-Related Perceptions of Airports by Residents—Development and Validation of the Aircraft Noise-Related Fairness Inventory (fAIR-In)
Source: Int J Environ Res Public Health. 2023 Jun 13;20(12):6113. doi: 10.3390/ijerph20126113 (PMC10297952; doi:10.3390/ijerph20126113)
Supplement: Supplementary file 1 [file ijerph-20-06113-s001.zip › ijerph-2392145-supplementary.pdf]

International Journal of
Environmental Research and Public Health

MDPI

Supplementary Material

1.1 Additional Tables

Table S1: Sample description according to the airport in the vicinity. CGN = Cologne-Bonn Airport, DUS = Dusseldorf Airport and DTM = Dortmund Airport

	CGN	DUS	DTM
	N (%)	N (%)	N (%)
Total	819 (59.9)	454 (33.2)	91 (6.7)
Gender			
male	455 (55.6)	287 (63.2)	64 (70.3)
female	359 (43.8)	163 (35.9)	27 (29.7)
diverse	5 (0.6)	4 (0.9)	0
Age			
18 - 24	33 (4)	16 (3.5)	2 (2.2)
25 - 34	130 (15.9)	60 (13.2)	13 (14.3)
35 - 44	158 (19.3)	67 (14.8)	19 (20.9)
45 - 54	150 (18.3)	84 (18.5)	14 (15.4)
55 - 64	208 (25.4)	124 (27.3)	26 (28.6)
65 - 74	98 (12)	67 (14.8)	16 (17.6)
75 - 84	39 (4.8)	30 (6.6)	1 (1.1)
>=85	3 (0.4)	6 (1.3)	0
Education			
still in school	5 (0.6)	2 (0.4)	0
primary education	35 (4.3)	17 (3.7)	3 (3.3)
lower secondary education	148 (18.1)	57 (12.6)	19 (20.9)
upper secondary education	631 (77)	378 (83.3)	69 (75.8
Living conditions			
renter	300 (36.6)	137 (30.2)	29 (31.9)
property owner	519 (63.4)	317 (69.8)	62 (68.1)
Job connected to airport			
direct	14 (1.7)	16 (3.5)	0
indirect	26 (3.2)	15 (3.3)	1 (1.1)
not connected	779 (95.1)	423 (93)	90 (98.9)
Noise exposure			
high exposure (>55 dB(A) L_{den})	452 (55.2)	313 (68.9)	73 (80.2)
low exposure (≤ 55 dB(A L_{den})	367 (44.8)	141 (31.1)	18 (19.8)
Participation			
online	782 (95.5)	436 (96)	88 (96.7)
paper-pencil	37 (4.5)	18 (4)	3 (3.3)

Table S2: Pearson r correlations between the individual fairness facets of the fAIR-In and all additional scales for testing construct validity separated according to the different degrees of noise exposure. (*** = $p \leq 0.001$, ** = $p \leq 0.01$, * = $p \leq 0.05$).

Constructs	Distributive Fairness		Procedural Fairness		Informational Fairness		Interpersonal Fairness	
Noise Exposure Level	low	high	low	high	low	high	low	high
Interpersonal Trust (KUSIV3)	-.08	.02	-.00	.04	.01	.06	.07	.06
Political Efficacy (PEKS)								
Internal political efficacy	-.10	-.03	-.18**	-.05	-.02	.04	-.11	-.08
External political efficacy	.12*	.25**	.20**	.29**	.26**	.31**	.23**	.33**
Injustice Sensitivity (USS-8)								
Victim sensitivity	.16*	.19**	.11	.10	.08	.04	.17*	.11*
Observer sensitivity	-.09	-.02	-.11	-.11*	-.06	-.06	-.01	.02
Beneficiary sensitivity	-.00	.05	-.06	.01	-.13*	.00	-.06	.03
Perpetrator sensitivity	-.14*	-.06	-.11	-.11*	-.09	-.06	-.10	-.04
Control Perception (IE-4)								
Internal control perception	-.16*	.02	-.13*	.08	.16**	.10*	.17**	.09
External control perception	.03	.04	.09	-.03	.04	-.07	.02	-.04
Political Cynicism (KPZ)	.04	-.20**	-.13*	-.22**	-.17**	-.27**	-.11	-.27**

Table S3: Pearson r correlations between the individual fairness facets of the fAIR-In and the predictive variables separated according to the different degrees of noise exposure. (*** = $p \leq 0.001$, ** = $p \leq 0.01$, * = $p \leq 0.05$).

Construct	Distributive Fairness		Procedural Fairness		Informational Fairness		Interpersonal Fairness	
Noise Exposure Level	low	high	low	high	low	high	low	high
Annoyance	-.71**	-.65**	-.64**	-.59**	-.50**	-.54**	-.61**	-.58**
Acceptance	.59**	.60**	.51**	.53**	.43**	.47**	.51**	.51**
Willingness to protest	-.43**	-.46**	-.33**	-.37**	-.20**	-.31**	-.36**	-.44**

1.2 fAIR-In Questionnaire in German with original items

Inventar zur Erfassung fluglärmbezogener Fairnesswahrnehmungen

fAIR-In

Dominik Hauptvogel, Dirk Schreckenberg, Tobias Rothmund, Marie-Therese Schmitz and Susanne Bartels

Zitation:

Hauptvogel, D., Schreckenberg, D., Rothmund, T., Schmitz, M.T., Bartels, S. (2023). Being a Fair Neighbor - Towards a Psychometric Inventory to Assess Fairness-Related Perceptions of the Airport by Residents -

Nutzungsbedinungen:

Dieses Erhebungsinstrument darf für nicht-kommerzielle Forschungszwecke kostenlos verwendet werden. Wird es für andere Zwecke oder in einer anderen als der hier dokumentierten Form verwendet, muss die Zustimmung der Autoren eingeholt werden. Bei allen daraus resultierenden Veröffentlichungen muss diese Arbeit als Quelle angegeben werden.

Inventar zur Erfassung fluglärmbezogener Fairnesswahrnehmungen (fAIR-In)

Im Folgenden lesen Sie einige Aussagen, die sich auf Aspekte der Nachbarschaftlichkeit beziehen. Geben Sie an, inwieweit die folgenden Aussagen Ihrer Ansicht nach stimmen oder nicht stimmen. Sollten Sie Schwierigkeiten haben, eine Frage zu beantworten, wählen Sie die Antwortmöglichkeit, die Ihrer Meinung nach am ehesten zutrifft. Es gibt keine richtigen und falschen Antworten. Uns ist Ihre ganz persönliche Meinung und Wahrnehmung wichtig.

		stimmt nicht	stimmt wenig	stimmt mittelmäßig	stimmt ziemlich	stimmt sehr
1	Der Flughafen bringt mir mehr Vorteile als Nachteile	1	2	3	4	5
2	Der Flughafen investiert ausreichend in Schallschutz, um die Anwohner/innen vor dem Lärm zu schützen.	1	2	3	4	5
3	Durch die unterschiedlichen An- und Abflugrichtungen der Flugzeuge wird die Lärmbelastung gleichmäßig auf die Anwohner/innen verteilt.	1	2	3	4	5
4	Die An- und Abflugrichtungen der Flugzeuge werden so variiert, dass alle Anwohner/innen auch einmal Zeiten der Ruhe genießen können.	1	2	3	4	5
5	Der Flughafen versucht, Anwohner/innen, die bereits von anderem Verkehrslärm (z.B. Straßen- oder Schienenlärm) stark betroffen sind, vor zusätzlicher Belastung durch Fluglärm zu schützen.	1	2	3	4	5
6	Der Flughafen bemüht sich den Fluglärm so zu verteilen, dass Naherholungsgebiete möglichst wenig von Fluglärm betroffen sind.	1	2	3	4	5
7	Die An- und Abflugrichtungen sind so gelegt, dass Schutzbedürftige, wie z.B. Kinder oder kranke Personen, möglichst wenig von Fluglärm betroffen sind.	1	2	3	4	5
8	Bevor Entscheidungen zum Fluglärm getroffen werden, habe ich die	1	2	3	4	5

	Möglichkeit, den Verantwortlichen meine Ansichten mitzuteilen.					
9	Als betroffene/r Anwohner/in werde ich in Entscheidungsprozesse des Flughafenmanagements einbezogen.	1	2	3	4	5
10	Der Flughafen geht in fluglärmrelevanten Entscheidungsprozessen aktiv auf seine Anwohner/innen zu, um deren Ansichten anzuhören.	1	2	3	4	5
11	Wenn Entscheidungen zum Fluglärm getroffen werden, kann ich auf die Ergebnisse des Entscheidungsprozesses Einfluss nehmen.	1	2	3	4	5
12	Als Anwohner/in werde ich bei Entscheidungen, die den Flughafen betreffen, vor vollendete Tatsachen gestellt.	1	2	3	4	5
13	Der Flughafen berücksichtigt bei Entscheidungsprozessen zum Fluglärm die Ansichten seiner Anwohner/innen.	1	2	3	4	5
14	Der Flughafen versucht, Entscheidungen unvoreingenommen und neutral zu treffen.	1	2	3	4	5
15	Bei Entscheidungen zum Fluglärm werden Informationen nur einseitig berücksichtigt.	1	2	3	4	5
16	Alle Parteien, die betroffen sind, werden bei fluglärmrelevanten Entscheidungen mit einbezogen.	1	2	3	4	5
17	In Entscheidungsprozessen zum Fluglärm, werden die Anliegen der betroffenen Anwohner/innen gut vertreten.	1	2	3	4	5
18	Es ist für Anwohner/innen nicht nachvollziehbar, wieso an unterschiedlichen Flughäfen unterschiedliche Regeln gelten, z.B. zu Nachtruhezeiten oder Flugverboten.	1	2	3	4	5

19	In den Entscheidungsprozessen fällen die Verantwortlichen Entscheidungen häufig auf der Basis von falschen Informationen.	1	2	3	4	5
20	Ich habe Möglichkeiten gegen getroffene Entscheidungen, die ich für falsch halte, vorzugehen.	1	2	3	4	5
21	Der Flughafen ist ehrlich bezüglich seiner Pläne für die Zukunft.	1	2	3	4	5
22	Informationen zum Thema Flugverkehr und Fluglärm werden vom Flughafen wahrheitsgemäß berichtet.	1	2	3	4	5
23	Der Flughafen erläutert und begründet fluglärmrelevante Entscheidungen ausführlich.	1	2	3	4	5
24	Der Flughafen kann mir verständlich erläutern, wie er versucht, Fluglärm zu vermeiden.	1	2	3	4	5
25	Wenn ich mich für das Thema Fluglärm interessiere, weiß ich, wo und wie ich durch den Flughafen weitere Informationen erhalte.	1	2	3	4	5
26	Der Flughafen bietet Anlaufstellen, an die ich mich wenden kann wenn ich etwas zum Thema Flugverkehr oder Fluglärm erfahren möchte.	1	2	3	4	5
27	Der Flughafen stellt Informationen bereit, die Anwohner/innen dazu befähigen, mit Lärmverantwortlichen auf Augenhöhe zu diskutieren.	1	2	3	4	5
28	Ich werde als Anwohner/in mit meinen Anliegen beim Flughafen ernst genommen.	1	2	3	4	5
29	Der Flughafen bemüht sich um einen Austausch mit lärmbetroffenen Anwohner/innen, der auf Augenhöhe geführt wird.	1	2	3	4	5

30	Der Flughafen zeigt aufrichtiges Verständnis für die Sorgen lärmbetroffener Anwohner/innen.	1	2	3	4	5
31	Der Flughafen versucht, auf die individuellen Bedürfnisse der Anwohner/innen einzugehen.	1	2	3	4	5
32	Der Austausch zwischen Flughafen und Anwohner/innen ist respektvoll.	1	2	3	4	5

1 Zusammenfassung

Der fAIR-In erhebt Aspekte der distributiven, prozeduralen, informationalen und interpersonalen Fairness in Bezug auf die Beziehung zwischen Flughafen und betroffenen AnwohnerInnen. Nicht-akustische Faktoren haben einen eherblichen Einfluss auf die Intensität der empfundenen Fluglärmlärmbelästigung. Fairness wurde als ein Faktor identifiziert, der vielen dieser nicht-akustischen Faktoren zugrunde liegt. Die Items des Fragebogens basieren auf Forschungsergebnissen zu Fairness aus anderen Kontexten (Adams, Leventhal, etc.) und auf Interviews mit von Fluglärm betroffenen AnwohnerInnen. Mit Hilfe des Fragebogens kann die Wahrnehmung des Flughafenmanagements aus Sicht der AnwohnerInnen erfasst und konkrete Ansatzpunkte für Interventionen mit dem Ziel, die Nachbarschaftlichkeit zwischen Flughafen und AnwohnerInnen zu verbesser, formuliert werden. Die Anwendung des Fragebogens vor, während und nach einer Interventionen ist zu empfehlen, um den aktuellen Stand, sowie den Erfolg nach einer durchgeführten Intervention zu erheben.

Antwortskala

5-Punkte Likert Scale: 1 = stimmt nicht, 2 = stimmt wenig, 3 = stimmt mittelmäßig, 4 = stimmt ziemlich, 5 = stimmt sehr

Werte nahe bei 5 deuten darauf hin, dass der Flughafen als fair empfunden wird. Werte nahe bei 1 deuten darauf hin, dass die Beziehungen zwischen dem Flughafen und den Anwohnern unzureichend sind.

Auswertungshinweise:

Für die Auswertung kann der Skalenwert (zwischen 1 und 5) für jedes beantwortete Item und jeden / jede Teilnehmer/in summiert und durch die Gesamtzahl der Antworten geteilt und dann gerundet werden.

Eine Teilanwendung und -auswertung auf der Ebene der Subskalen ist zulässig, da es sich um reliable und valide Subskalen handelt. Bitte beachten Sie die entsprechende Itemzuordnung.

Hinweis:

In der Fairness-Forschung wurden die verschiedenen Subfacetten als unabhängige Faktoren identifiziert, sie stehen jedoch in einer wechselseitigen Beziehung zueinander. Um ein faires, nachbarschaftliches

Verhältnis zu den BewohnerInnen aufzubauen, ist daher ein ganzheitlicher Ansatz erforderlich, der alle Facetten gleichermaßen berücksichtigt.

Umsetzung der Testergebnisse im Flughafenmanagement:
Für die Umsetzung der Ergebnisse im Sinne eines fairen, nachbarschaftlichen Umgangs gibt es keine grundsätzlichen Empfehlungen und es müssen je nach Kontext und Charakteristik des Flughafens individuelle Entscheidungen getroffen werden.

Es lassen sich jedoch Grundprinzipien ableiten, die sich auf die aktuelle Fairness-Forschung beziehen. Eine Übersicht und Diskussion möglicher Interventionen, die individuelle Fairness-Aspekte einbeziehen, findet sich in dem Review von Hauptvogel et al. (2021).

1.3 fAIR-In Questionnaire translated into English

Aircraft Noise related Fairness Inventory (fAIR-In)

Aircraft Noise related Fairness Inventory

fAIR-In

Dominik Hauptvogel, Dirk Schreckenberg, Tobias Rothmund, Marie-Therese Schmitz and Susanne Bartels

Citation:

Hauptvogel, D., Schreckenberg, D., Rothmund, T., Schmitz, M.T., Bartels, S. (2023). Being a Fair Neighbor - Towards a Psychometric Inventory to Assess Fairness-Related Perceptions of the Airport by Residents - Development and Validation of the Aircraft Noise related Fairness Inventory (fAIR-In). Int. J. Environ. Res. Public Health 2023

Terms of use:

This survey instrument may be used free of charge for non-commercial research purposes. If it is used for other purposes or in a form other than that documented here, the consent of the of the authors must be obtained. In all resulting publications, this documentation must be cited

as the source.

Below you will read several statements that relate to aspects of neighborliness.
Please indicate to what extent you think the following statements are true or not true.
If you have difficulty answering a question, choose the answer option that you think is most appropriate.
There are no right and wrong answers. Your personal opinion and perception are important to us.

		Not true	A Little true	Moderately true	Quite a bit true	Very true
1	The airport brings me more advantages than disadvantages.	1	2	3	4	5
2	The airport invests sufficiently in noise protection to protect residents from the noise.	1	2	3	4	5
3	Due to the different approach and departure routes of the aircraft, the noise exposure is evenly distributed among the residents.	1	2	3	4	5
4	The approach and departure routes of the aircraft are varied in such a way that all residents can also enjoy periods of peace and quiet.	1	2	3	4	5
5	The airport attempts to protect residents who are already affected by other traffic noise (e.g. road or rail noise) from further exposure to aircraft noise.	1	2	3	4	5
6	The airport strives to distribute aircraft noise in such a way that local recreation areas are affected as little as possible by aircraft noise.	1	2	3	4	5
7	The approach and departure routes are designed in such a way that those in need of special protection, such as children or sick individuals, are affected as little as possible by aircraft noise.	1	2	3	4	5
8	Before decisions are made on aircraft noise, I have the opportunity to make my views known to those responsible.	1	2	3	4	5

9	As an affected resident, I am involved in the decision-making process of the airport management.	1	2	3	4	5
10	The airport actively approaches its residents in decision-making processes regarding aircraft noise to listen to their opinions.	1	2	3	4	5
11	When decisions are made about aircraft noise, I can influence the outcome of the decision-making process.	1	2	3	4	5
12	As a resident, I am presented with a fait accompli when it comes to decisions concerning the airport.	1	2	3	4	5
13	The airport takes the views of its residents into account in decision-making processes regarding aircraft noise.	1	2	3	4	5
14	The airport attempts to make decisions in an impartial and neutral manner.	1	2	3	4	5
15	In decisions on aircraft noise, information is only taken into account from one perspective.	1	2	3	4	5
16	All affected parties are involved in decisions regarding aircraft noise.	1	2	3	4	5
17	In decision-making processes regarding aircraft noise, the concerns of affected residents are well represented.	1	2	3	4	5
18	It is not understandable for residents why different rules apply at different airports, e.g. on night curfews or flight bans.	1	2	3	4	5
19	In the decision-making processes, those in charge often reach decisions on the basis of incorrect information.	1	2	3	4	5
20	I have possibilities to take action against decisions that I think are wrong.	1	2	3	4	5
21	The airport is honest about its plans for the future.	1	2	3	4	5

22	Information on air traffic and aircraft noise is reported truthfully by the airport.	1	2	3	4	5
23	The airport explains and justifies decisions relevant to aircraft noise in detail.	1	2	3	4	5
24	The airport can explain to me in an understandable way how it tries to avoid aircraft noise.	1	2	3	4	5
25	If I am interested in the topic of aircraft noise, I know where and how to get more information through the airport.	1	2	3	4	5
26	The airport offers contact points that I can turn to if I want to know something about air traffic or aircraft noise.	1	2	3	4	5
27	The airport provides information that enables residents to discuss with noise authorities at eye level.	1	2	3	4	5
28	As a resident, my concerns are taken seriously by the airport.	1	2	3	4	5
29	The airport strives for an exchange with noise-affected residents that is conducted on an equal footing.	1	2	3	4	5
30	The airport shows genuine understanding for the concerns of residents affected by noise.	1	2	3	4	5
31	The airport tries to respond to the individual needs of the residents.	1	2	3	4	5
32	The exchange between airport and residents is respectful.	1	2	3	4	5

Note: Rating scale was translated from German into English according to Rohrmann (2007; [1]) and can be seen as approximately interval scaled.

1 Summary

The fAIR-In measures aspects of distributive, procedural, informational and interpersonal fairness in relation to the relationship between the airport and affected residents.
Non-acoustic factors have a significant impact on the intensity of aircraft noise annoyance experienced.
Fairness was identified as an underlying factor of a number of non-acoustic factors.
The items of the questionnaire are based on research on fairness in other contexts (Adams, Leventhal, etc.) and on interviews with residents affected by aircraft noise.
With the help of the questionnaire, the perception of the airport management from the residents' point of view can be captured, and concrete starting points for interventions can be formulated with the aim of increasing the neighborliness between airport and residents. It is helpful to use the questionnaire during an intervention, as well as in pre- and post-comparison to measure the success of interventions.

Response specifications

5-point Likert Scale: 1 = not true, 2 = a little true, 3 = moderately true, 4 = quite a bit true, 5 = very true
Values close to 5 indicate a perception of the airport as being fair. Values close to 1 indicate that there are deficiencies in the relationship between the airport and residents.

Evaluation instructions:

For evaluation, the scale value (between 1 and 5) for each answered item and respondent can be summed and divided by the total number of responses and then rounded.
Partial application and evaluation is acceptable at the subscale level, as these are reliable and valid subscales. Please note the corresponding item assignment.

Note:

In research on fairness, the various sub-facets have been identified as independent factors, but they are mutually interrelated. A holistic approach is therefore needed to establish a fair, neighborly relationship with residents that takes all facets into account equally.

Implementation of the test results in airport management:

For the implementation of the results in the sense of a fair, neighborly relationship, there are no basic recommendations and individual decisions must be taken depending on the context and characteristics of the airport.

However, basic principles can be derived that relate to current research on fairness.
An overview and discussion of potential interventions that include individual fairness aspects can be found in the review by Hauptvogel et al. (2021).

1.4 fAIR-In Items in German and English with classification to facts and subfacets

Facet description

D = Distributive
P = Procedural
IF = Informational
IP = Interpersonal

Nr	Item in German	Items in English	Polarisation	Facette	Subfacette
1	Der Flughafen bringt mir mehr Vorteile als Nachteile	The airport brings me more advantages than disadvantages.	+	D	Equity
2	Der Flughafen investiert ausreichend in Schallschutz, um die Anwohner/innen vor dem Lärm zu schützen.	The airport invests sufficiently in noise protection to protect residents from the noise.	+	D	Equity
3	Durch die unterschiedlichen An- und Abflugrichtungen der Flugzeuge wird die Lärmbelastung gleichmäßig auf die Anwohner/innen verteilt.	Due to the different approach and departure routes of the aircraft, the noise exposure is evenly distributed among the residents.	+	D	Equality
4	Die An- und Abflugrichtungen der Flugzeuge werden so variiert, dass alle Anwohner/innen auch einmal Zeiten der Ruhe genießen können.	The approach and departure routes of the aircraft are varied in such a way that all residents can also enjoy periods of peace and quiet.	+	D	Equality
5	Der Flughafen versucht, Anwohner/innen, die bereits von anderem Verkehrslärm (z.B. Straßen- oder Schienenlärm) stark betroffen sind, vor zusätzlicher Belastung	The airport attempts to protect residents who are already affected by other traffic noise (e.g. road or rail noise) from further exposure to aircraft noise.	+	D	Need

	durch Fluglärm zu schützen.				
6	Der Flughafen bemüht sich den Fluglärm so zu verteilen, dass Naherholungsgebiete möglichst wenig von Fluglärm betroffen sind.	The airport strives to distribute aircraft noise in such a way that local recreation areas are affected as little as possible by aircraft noise.	+	D	Need
7	Die An- und Abflugrichtungen sind so gelegt, dass Schutzbedürftige, wie z.B. Kinder oder kranke Personen, möglichst wenig von Fluglärm betroffen sind.	The approach and departure routes are designed in such a way that those in need of special protection, such as children or sick individuals, are affected as little as possible by aircraft noise.	+	D	Need
8	Bevor Entscheidungen zum Fluglärm getroffen werden, habe ich die Möglichkeit, den Verantwortlichen meine Ansichten mitzuteilen.	Before decisions are made on aircraft noise, I have the opportunity to make my views known to those responsible.	+	P	Process Control
9	Als betroffene/r Anwohner/in werde ich in Entscheidungsprozesse des Flughafenmanagements einbezogen.	As an affected resident, I am involved in the decision-making process of the airport management.	+	P	Process Control
10	Der Flughafen geht in fluglärmrelevanten Entscheidungsprozessen aktiv auf seine Anwohner/innen zu, um deren Ansichten anzuhören.	The airport actively approaches its residents in decision-making processes regarding aircraft noise to listen to their opinions.	+	P	Process Control
11	Wenn Entscheidungen zum Fluglärm getroffen	When decisions are made about aircraft	+	P	Decision Control

	werden, kann ich auf die Ergebnisse des Entscheidungsprozesses Einfluss nehmen.	noise, I can influence the outcome of the decision-making process.			
12	Als Anwohner/in werde ich bei Entscheidungen, die den Flughafen betreffen, vor vollendete Tatsachen gestellt.	As a resident, I am presented with a fait accompli when it comes to decisions concerning the airport.	-	P	Decision Control
13	Der Flughafen berücksichtigt bei Entscheidungsprozessen zum Fluglärm die Ansichten seiner Anwohner/innen.	The airport takes the views of its residents into account in decision-making processes regarding aircraft noise.	+	P	Decision Control
14	Der Flughafen versucht, Entscheidungen unvoreingenommen und neutral zu treffen.	The airport attempts to make decisions in an impartial and neutral manner.	+	P	Bias Supression
15	Bei Entscheidungen zum Fluglärm werden Informationen nur einseitig berücksichtigt.	In decisions on aircraft noise, information is only taken into account from one perspective.	-	P	Bias Supression
16	Alle Parteien, die betroffen sind, werden bei fluglärmrelevanten Entscheidungen mit einbezogen.	All affected parties are involved in decisions regarding aircraft noise.	+	P	Represent-ativeness
17	In Entscheidungsprozessen zum Fluglärm, werden die Anliegen der betroffenen Anwohner/innen gut vertreten.	In decision-making processes regarding aircraft noise, the concerns of affected residents are well represented.	+	P	Represent-ativeness
18	Es ist für Anwohner/innen nicht nachvollziehbar, wieso an unterschiedlichen	It is not understandable for residents why different rules apply at different airports, e.g.	-	P	Consistency

	Flughäfen unterschiedliche Regeln gelten, z.B. zu Nachtruhezeiten oder Flugverboten.	on night curfews or flight bans.			
19	In den Entscheidungsprozessen fällen die Verantwortlichen Entscheidungen häufig auf der Basis von falschen Informationen.	In the decision-making processes, those in charge often reach decisions on the basis of incorrect information.	-	P	Accuracy
20	Ich habe Möglichkeiten gegen getroffene Entscheidungen, die ich für falsch halte, vorzugehen.	I have possibilities to take action against decisions that I think are wrong.	+	P	Correct- ability
21	Der Flughafen ist ehrlich bezüglich seiner Pläne für die Zukunft.	The airport is honest about its plans for the future.	+	IF	Truth- fulness
22	Informationen zum Thema Flugverkehr und Fluglärm werden vom Flughafen wahrheitsgemäß berichtet.	Information on air traffic and aircraft noise is reported truthfully by the airport.	+	IF	Truth- fulness
23	Der Flughafen erläutert und begründet fluglärmrelevante Entscheidungen ausführlich.	The airport explains and justifies decisions relevant to aircraft noise in detail.	+	IF	Justification
24	Der Flughafen kann mir verständlich erläutern, wie er versucht, Fluglärm zu vermeiden.	The airport can explain to me in an understandable way how it tries to avoid aircraft noise.	+	IF	Justification
25	Wenn ich mich für das Thema Fluglärm interessiere, weiß ich,	If I am interested in the topic of aircraft noise, I know where and how	+	IF	Empower- ment

	wo und wie ich durch den Flughafen weitere Informationen erhalte.	to get more information through the airport.			
26	Der Flughafen bietet Anlaufstellen, an die ich mich wenden kann wenn ich etwas zum Thema Flugverkehr oder Fluglärm erfahren möchte.	The airport offers contact points that I can turn to if I want to know something about air traffic or aircraft noise.	+	IF	Empower- ment
27	Der Flughafen stellt Informationen bereit, die Anwohner/innen dazu befähigen, mit Lärmverantwortlichen auf Augenhöhe zu diskutieren.	The airport provides information that enables residents to discuss with noise authorities at eye level.	+	IF	Empower- ment
28	Ich werde als Anwohner/in mit meinen Anliegen beim Flughafen ernst genommen.	As a resident, my concerns are taken seriously by the airport.	+	IP	Propriety
29	Der Flughafen bemüht sich um einen Austausch mit lärmbetroffenen Anwohner/innen, der auf Augenhöhe geführt wird.	The airport strives for an exchange with noise-affected residents that is conducted on an equal footing.	+	IP	Propriety
30	Der Flughafen zeigt aufrichtiges Verständnis für die Sorgen lärmbetroffener Anwohner/innen.	The airport shows genuine understanding for the concerns of residents affected by noise.	+	IP	Propriety
31	Der Flughafen versucht, auf die individuellen Bedürfnisse der Anwohner/innen einzugehen.	The airport tries to respond to the individual needs of the residents.	+	IP	Respect

32	Der Austausch zwischen Flughafen und Anwohner/innen ist respektvoll.	The exchange between airport and residents is respectful.	+	IP	Respect

1.5 Additional Scales used to measure predictive validity in this study

Table S4: Questionnaire used in this study to measure acceptance of the airport and air travel.

Questioning	Items in German	Items in English	Polarization	Answers
Ich halte den Flugverkehr ganz allgemein für...	notwendig	necessary	+	(1) Stimmt nicht<br>(2) Stimmt wenig<br>(3) Stimmt mittelmäßig<br>(4) Stimmt ziemlich<br>(5) Stimmt sehr
	gesundheitsgefährend	harmful	-	
	unsicher	unsafe	-	
I consider air traffic in general to be..	umweltschädlich	Environmentally damaging	-	(1) Not true<br>(2) A little true<br>(3) Moderately true<br>(4) Quite a bit true<br>(5) Very true
	vermeidbar	avoidable	-	
	schlecht für die Luftqualität	Bad for air quality	-	
	klimaschädlich	climate damaging	-	

Table S5: Questionnaire used in this study to measure protest behaviour.

Questioning	Items in German	Items in English	Answers
Haben/Sind Sie im Zusammenhang mit der Fluglärmthematik...<br>In connection with the aircraft noise topic, have you…	... eine Protestliste, Petition oder Ähnliches unterschrieben?	... signed a protest list, petition or similar?	(0) Nein<br>(1) Ja<br>Wert ergibt sich aus der Summe positiv beantworteter Aussagen.<br>(0) No<br>(1) Yes<br>Value results from the sum of positively answered statements
	... Kontakt zum Flughafen oder einer zuständigen Stelle aufgenommen, um Informationen zu erhalten?	... contacted the airport or a responsible office for information?	
	... Kontakt zum Flughafen oder einer zusändigen Stelle aufgenommen, um sich zu beschweren?	... Have you contacted the airport or a competent authority to complain?	
	... einer Bürgerinitiative gegen Fluglärm beigetreten?	... joined a citizens' initiative against aircraft noise?	
	... an einer Demonstration teilgenommen?	... participated in a demonstration?	
	... in ein anderes Gebiet gezogen?	... moved to another area?	
	(falls nein) Ziehen Sie einen Umzug in Betracht?	(if no) are you considering moving?	

References

1. Rohrmann, B. Verbal qualifiers for rating scales: Sociolinguistic considerations and psychometric data. *Project Report, University of Melbourne/Australia* **2007**.
